# Hydrogenation and hydrodeoxygenation of biomass-derived oxygenates to liquid alkanes for transportation fuels

**DOI:** 10.1016/j.dib.2018.01.069

**Published:** 2018-01-31

**Authors:** Shaohui Sun, Ruishu Yang, Xin Wang, Shaokang Yan

**Affiliations:** Research Center of Heterogeneous Catalysis & Engineering Science, School of Chemical Engineering and Energy, Zhengzhou University, Zhengzhou City, China

## Abstract

An attractive approach for the production of transportation fuels from renewable biomass resources is to convert oxygenates into alkanes. In this paper, C_5_–C_20_ alkanes formed via the hydrogenation and hydrodeoxygenation of the oligomers of furfuryl alcohol(FA) can be used as gasoline, diesel and jet fuel fraction. The first step of the process is the oligomers of FA convert into hydrogenated products over Raney Ni catalyst in a batch reactor. The second step of the process converts hydrogenated products to alkanes via hydrodeoxygenation over different bi-functional catalysts include hydrogenation and acidic deoxidization active sites. After this process, the oxygen content decreased from 22.1 wt% in the oligomers of FA to 0.58 wt% in the hydrodeoxygenation products.

**Specifications Table**

**Table:** 

Subject area	Biomass
More specific subject area	Bioenergy
Type of data	Table, figure
How data was acquired	Gas chromatography-mass spectrometry (GC–MS), gas chromatography (GC), Fourier Transform infrared spectroscopy(FTIR), X-ray diffraction (XRD).
Data format	Analyzed
Experimental factors	Prepared long chain alkanes with FA condensate as raw materials.
Experimental features	Content data gathered after the run of the reaction
Data source location	Zhengzhou, China
Data accessibility	The data are with this article

**Value of the data**

• This data shows the theoretical content of C, H, O in the oligomers of FA and elemental analysis of hydrogenation products.
• This data provides the result of the hydrogenation products and hydrodeoxygenation products GC–MS spectra, structural formula and peak flowing out time.
• This data given the power X-ray diffraction (XRD) patterns of fresh catalysts and there average particle sizes of metal site.
• This data provides simulated distillation curves for different high-boiling components in the hydrodeoxygenation products.

## 1 Data

**Fig. 1:**
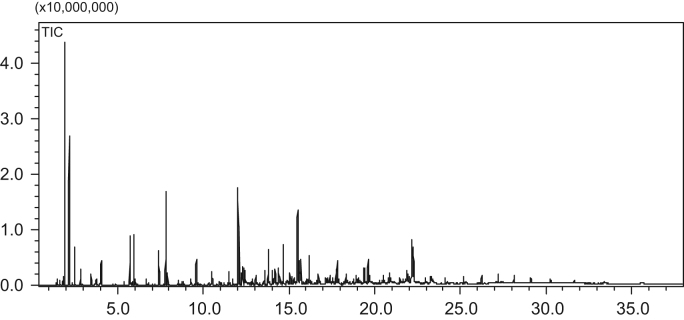
The GC–MS chromatogram of hydrogenation products in reaction conditions of 12 g FA condensation products, 5 g Raney Ni, 60 g THF, 6 MPa H_2_, reaction 4 h.

**Fig. 2:**
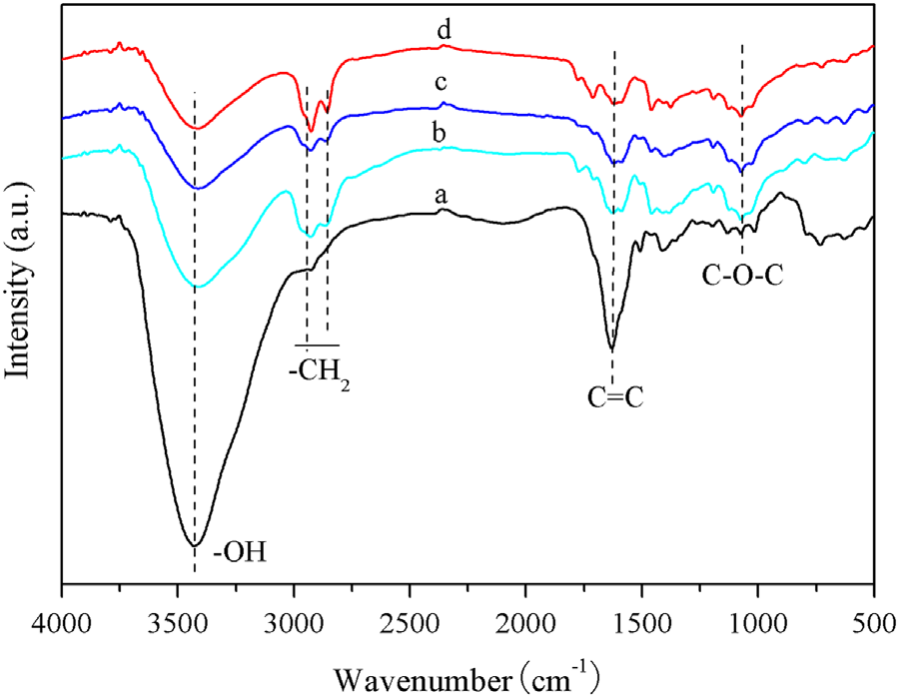
FT-IR spectra of the oligomers of FA and hydrogenation products in reaction conditions of 12 g oligomers of FA, 5 g Raney Ni, 60 g THF, 6 MPa H_2_, reaction 4 h in a batch reactor of 300 ml at different temperatures, (a)the oligomers of FA; (b)110 °C; (c)130 °C; (d)150 °C.

**Fig. 3:**
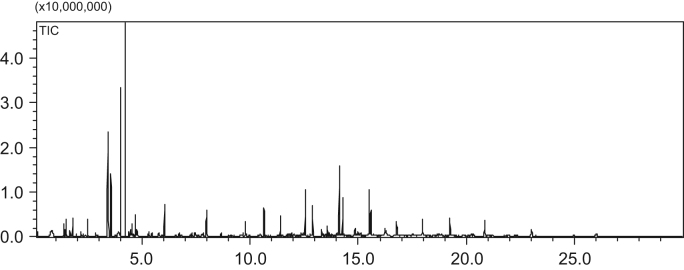
The GC–MS chromatogram of hydrodeoxygenation products in reaction conditions of 5.7 g hydrogenation products, 2.6 g 5 Wt% Pd/H-ZSM-5, 80 g n-octane, 6 MPa H_2_,170 °C, reaction 5 h in a batch reactor of 300 ml.

**Fig. 4:**
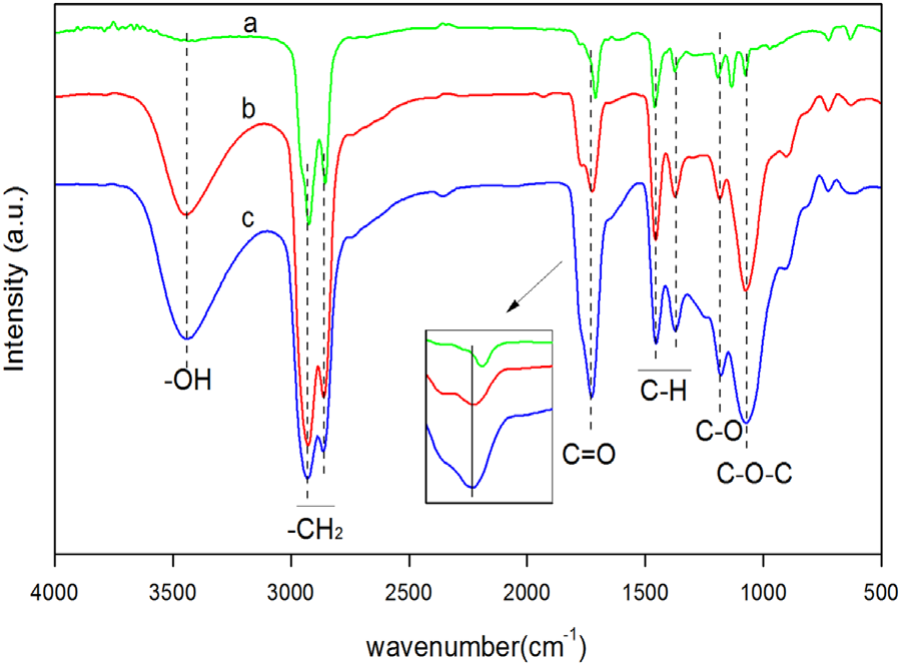
FT-IR spectra of high-boiling component in the hydrodeoxygenation products in first step reaction conditions of 12 g the oligomers of FA, 5 g Raney Ni, 60 g THF, 6 MPa H_2_, reaction 4 h at different temperatures and in second step reaction conditions of 5.7 g hydrogenation products, 2.6 g 5 wt% Pd/H-ZSM-5, 80 g n-octane, 6 MPa H_2_, reaction 5 h at different temperatures in a batch reactor of 300 ml, (a) first step temperature 130 °C, second step temperature 170 °C; (b) first step temperature 150 °C, second step temperature 150 °C; (c) first step temperature 130 °C, second step temperature 150 °C.

**Fig. 5:**
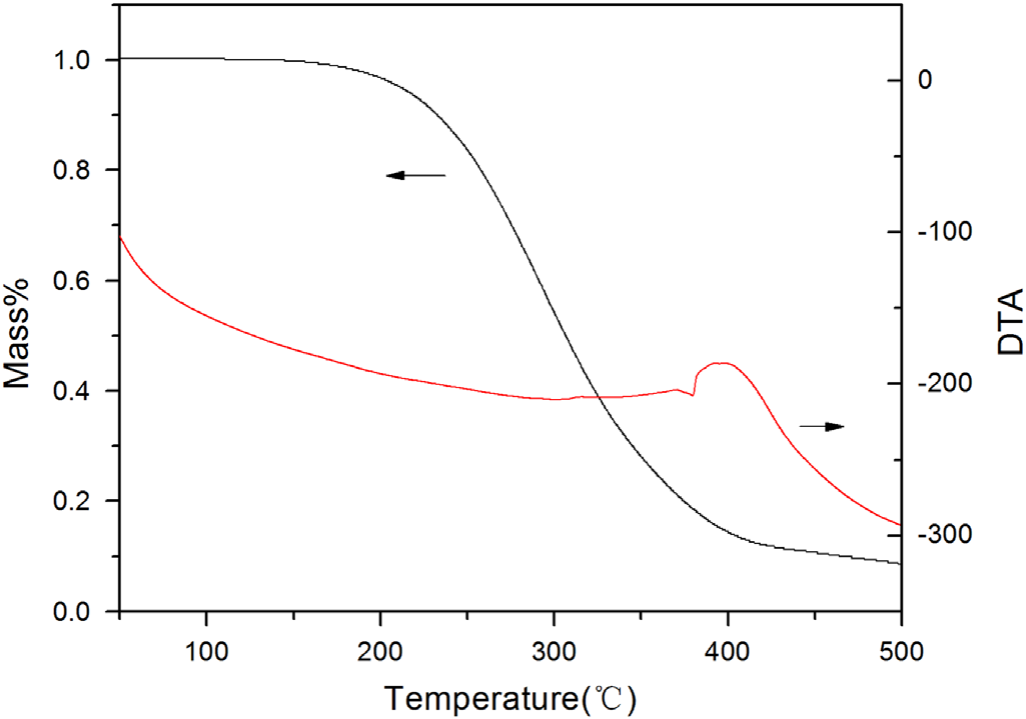
The DTA-TGA curve of high-boiling components in the hydrodeoxygenation products in reaction conditions of 5.7 g hydrogenation products, 2.6 g 5 Wt% Pd/H-ZSM-5, 80 g n-octane, 6 MPa H_2_,170 °C, reaction 5 h in a batch reactor of 300 ml.

**Fig. 6:**
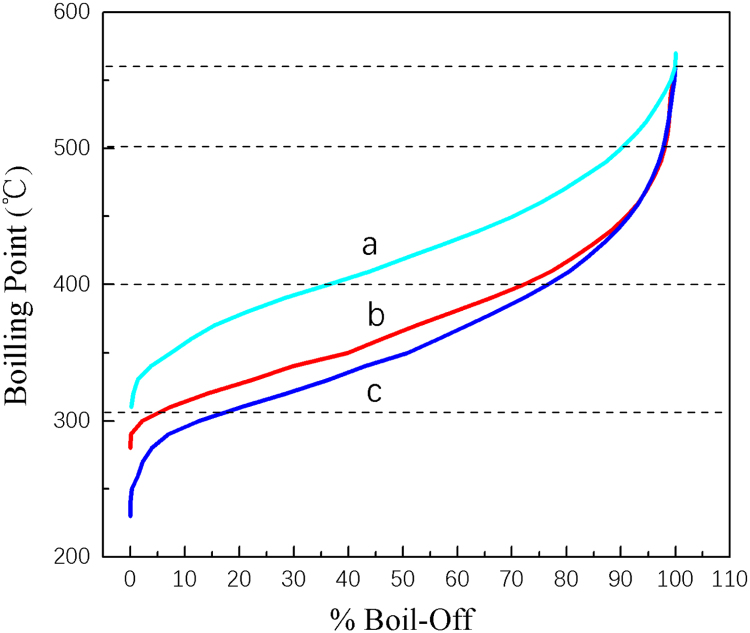
Simulated distillation curves for different high-boiling components in the hydrodeoxygenation products in first step reaction conditions of 12 g the oligomers of FA, 5 g Raney Ni, 60 g THF, 6 MPa H_2_, reaction 4 h at different temperatures and in second step reaction conditions of 5.7 g hydrogenation products, 2.6 g 5 Wt% Pd/H-ZSM-5, 80 g n-octane, 6 MPa H_2_, reaction 5 h at different temperatures in a batch reactor of 300 ml, (a) first step temperature 130 °C, second step temperature 170 °C; (b) first step temperature 150 °C, second step temperature 170 °C; (c) first step temperature 150 °C, second step temperature 150 °C.

**Fig. 7:**
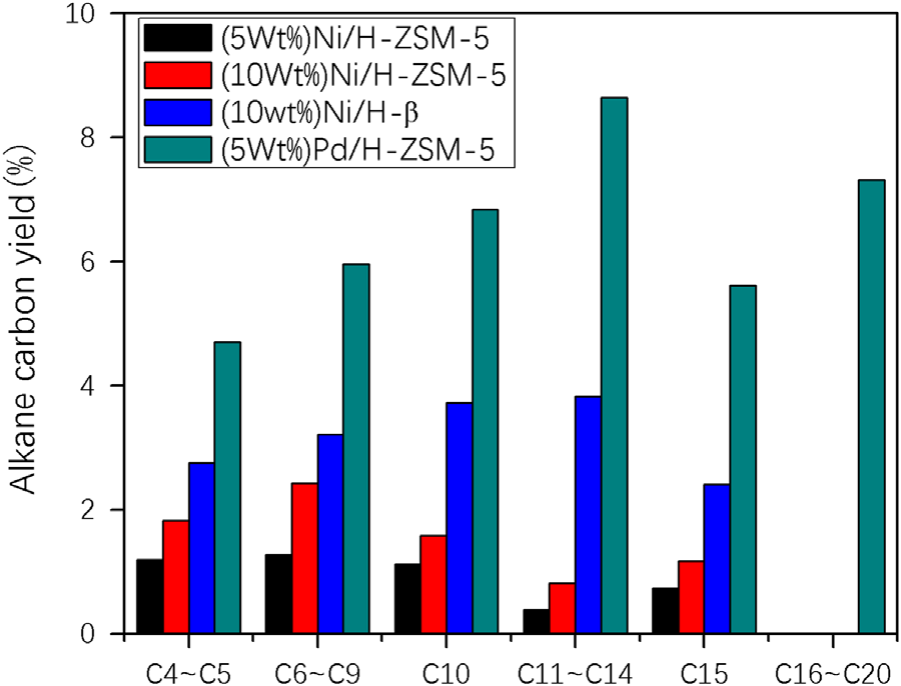
The carbon yield of different alkanes in the hydrodeoxygenation products over different catalysts in reaction conditions of 2.6 g catalysts, 80 g n-octane, 6 MPa H_2_, 170 °C, reaction 5 h in a batch reactor of 300 ml.

**Fig. 8:**
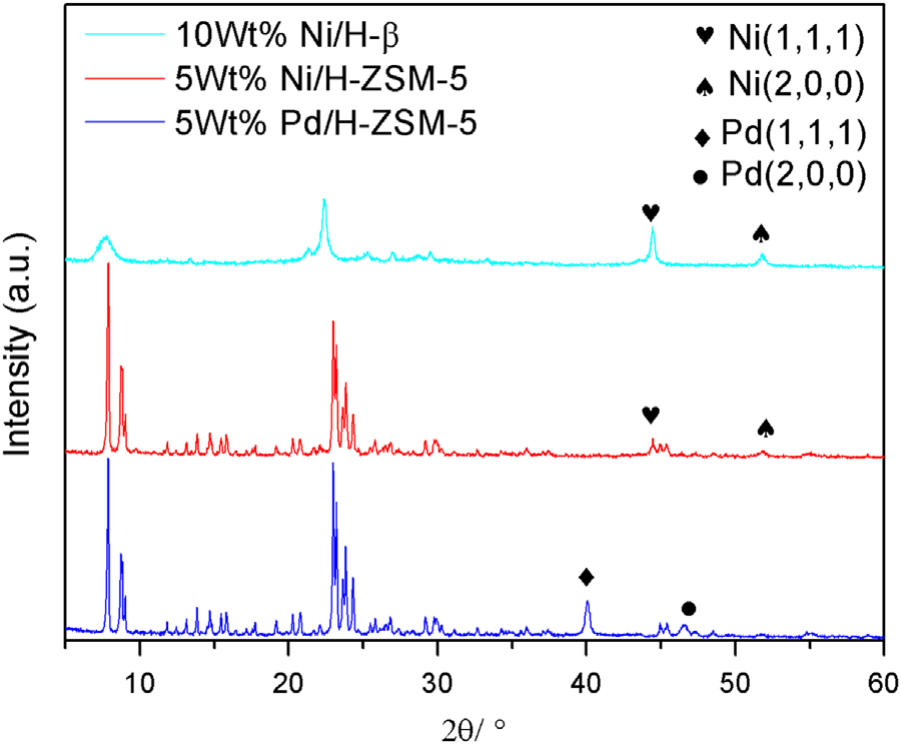
Power X-ray diffraction patterns of fresh catalysts.

The data in this data article has been gathered under a “The study of carbon-carbon bond forming of biomass-derived furfuryl alcohol by self-condensation and subsequent hydrogenation 21376226” run under the National Natural Science Foundation of China program funded by the state government of China. Table 1 shows the result analysis of hydrogenation products over different reaction temperature. Table 2 shows the result of hydrogenation products by GC–MS analysis. Table 3 shows the theoretical content of C, H, O in the oligomers of FA and elemental analysis of hydrogenation products. Table 4 shows the alkane carbon yield and TOF under different catalysts. Fig. 1 shows the GC–MS chromatogram of hydrogenation products in reaction conditions of 12 g oligomers of FA, 5 g Raney Ni, 60 g THF, 6 MPa H_2_, reaction 4 h. Fig. 2 shows FT-IR spectra of the oligomers of FA and hydrogenation products in reaction conditions of 12 g oligomers of FA, 5 g Raney Ni, 60 g THF, 6 MPa H_2_, reaction 4 h at different temperatures,(a)the oligomers of FA; (b)110 °C; (c)130 °C; (d)150 °C. Fig. 3 shows power X-ray diffraction patterns of fresh catalysts. Table 5 shows average particle sizes of metal site in different catalysts (Fig. 4, Fig. 5, Fig. 6, Fig. 7, Fig. 8 and Table 6).

**Table 1:** The result of hydrogenation products over different reaction temperature.^a^

Reaction temperature °C	The H_2_ consumption (mol)	The mass of hydrogenation products (g)	Carbons distribution C_5_–C_20_ (%)^b^			
			C_5_	C_6_–C_10_	C_11_–C_15_	C_16_–C_20_
130	0.17	9.27	4.27	30.01	32.18	33.54
150	0.24	8.57	4.94	38.32	34.21	22.53

a Conditions: 12 g oligomers of FA, 5 g Raney Ni, 60 g tetrahydrofuran (THF), 6 MPa H_2_, reaction 4 h in a batch reactor of 300 mL.

b Relative peak area.

**Table 2:** The result of hydrogenation products by GC–MS analysis.

**Table 3:** The theoretical content of C, H, O in the oligomers of FA and elemental analysis of hydrogenation products.

Run	Raw material	C (wt%)	H (wt%)	O (wt%)	H/C
1	The oligomers of FA	72.7	5.15	22.1	0.85
2	Hydrogenation products^a^	65.6	11.6	22.8	2.13
3	Hydrogenation products^b^	67.6	12.5	19.9	2.22

a Conditions: 12 g the oligomers of FA, 5 g Raney Ni, 60 g THF, 6 MPa H_2_,130 °C, reaction 4 h in a batch reactor of 300 mL.

b Conditions: 12 g the oligomers of FA, 5 g Raney Ni, 60 g THF, 6 MPa H_2_,150 °C, reaction 4 h in a batch reactor of 300 mL.

**Table 4:** The result of GC–MS analysis of hydrodeoxygenation products in reaction conditions of 5.7 g hydrogenation products, 2.6 g 5 wt% Pd/H-ZSM-5, 80 g n-octane, 6 MPa H_2_,170 °C, reaction 5 h in a batch reactor of 300 mL.

**Table 5:** The alkane carbon yield and TOF under different catalysts in reaction conditions of 2.6 g catalysts, 80 g n-octane, 6 MPa H_2_, 170 °C, reaction 5 h in a batch reactor of 300 mL.

Run	Hydrogenated products (g)^a^	Catalyst	The components of boiling temperature < 300 °C				The components of boiling temperature > 300 °C	
			Mass (g)	Alkane carbon yield (%)	C_9_–C_20_ yield (%)	TOF (mol g^−1^ h^−1^)	Mass (g)	O (wt%)
1	5.70	5 wt% Pd/H-ZSM-5	1.90	39.1	31.2	1.40	0.95	0.59
2	8.20	5 wt% Ni/H-ZSM-5	0.33	4.72	2.88	0.24	0	0
3	7.47	10 wt% Ni/ H-β	1.02	15.9	12.1	0.66	1.38	8.62
4	6.96	10 wt% Ni/HZSM-5	0.47	7.83	4.73	0.17	1.52	12.4
5	7.61	5 wt% Pt/C+H-ZSM-5^b^	0	0	0	0	1.67	14.4
6	7.50	5 wt% Ni/Al_2_O_3_	0	0	0	0	1.49	14.22

a Conditions: 12 g the oligomers of FA, 5 g Raney Ni, 60 g THF, 6 MPa H_2_,130 °C, reaction 4 h in a batch reactor of 300 mL.

b Physical mixing the C and H-ZSM-5.

**Table 6:** Average particle sizes of metal site in different catalysts.

Run	Catalyst	Average particle size of metal (nm)^a^
1	5 wt% Pd/H-ZSM-5	6.34
2	5 wt% Ni/H-ZSM-5	6.15
3	10 wt% Ni/H-β	3.57

a The values of average particle size of metal have been calculated from Debye-Scherrer formula.

## 2 Experimental design, materials and methods

### 2.1 FA condensation reaction

Oligomerization of FA was performed with an aqueous solution containing FA (50 g) and 1.25 wt% sulfuric acid (150 g) at 50 °C and reaction 30 min in a three-necked flask of 500 ml, stirring at 300 r min^−1^, and put nitrogen into flask during the reaction [1]. After centrifugal separation, the unreacted FA in the organic phase was washed with water several times to obtain a monomer-free condensation products required in the next step.

### 2.2 Hydrogenation

The condensation products in THF were reacted under hydrogen pressure of 6 MPa at different temperature (130–150 °C) over Raney Ni catalyst in a batch reactor of 300 ml, stirring at 600 r min^−1^ and reaction 4 h.

### 2.3 Hydrodeoxygenation

The products of the hydrogenation were reacted under hydrogen pressure of 6 MPa at 170 °C in a batch reactor of 300 ml, stirring at 600 r min^−1^ and reaction 5 h over different bi-functional catalysts, which were prepared with an incipient wetness impregnation method.

### 2.4 Analytical techniques

GC chromatographic conditions: A gas chromatograph SHIMAPZU GC-14B equipped with a flame ionization detector (FID) and Agilent DB-5 capillary column (0.25 mm*0.25 µm, 30 m). The GC temperature profile used was as follows: injector Temp. 250 °C; FID Temp., 250 °C; oven Temp., 100 °C held for 3 min, ramp rate, 6 °C min^−1^, and final Temp., 300 °C held for 3 min. An injection volume of 0.1 µL was employed. High purity nitrogen was used as the carrier gas at a flow rate of 1.0 mL min^−1^. The split ratio was set to 30:1.

GC/MS chromatographic conditions: Capillary chromatography equipped with SHIMAPZU GC-2010 capillary chromatograph. Chromatographic operating conditions same with before except the carrier gas is high purity helium with a split ratio of 80:1.

GC/MS mass spectrometry conditions: Electron impact ion source: 70 eV; ion source temperature: 250 °C; scanning range (m/z): 30 u–500 amu; scanning mode is for full scan monitoring.

Functional groups in the oligomers of FA, hydrogenation products and hydrodeoxygenation products, and the changes in these groups were determined by Nicolet 6700 infrared absorption spectrometer from Thermo Fisher Scientific (FTIR). The sweep frequency range is 400–4000 cm^−1^; The resolution is 2 cm^−1^, and the scanning frequency is 32 times.

Carbon, hydrogen and oxygen elemental content of the organic samples were determined using the Flash EA 1112 element analyzer from Thermo Electron SPA.
